# Imbalanced weighting of proactive and reactive control as a marker of risk-taking propensity

**DOI:** 10.1371/journal.pone.0277246

**Published:** 2023-01-20

**Authors:** Fanny Grisetto, Pierre Le Denmat, Yvonne N. Delevoye-Turrell, Quentin Vantrepotte, Tanguy Davin, Andreea Dinca, Isabelle Desenclos-El Ghoulti, Clémence Roger

**Affiliations:** 1 Univ. Lille, CNRS, UMR 9193—SCALab—Sciences Cognitives et Sciences Affectives, Lille, France; 2 ECCA Conduite, Lyon, France; University of Sydney, AUSTRALIA

## Abstract

According to the dual mechanisms of control (DMC), reactive and proactive control are involved in adjusting behaviors when maladapted to the environment. However, both contextual and inter-individual factors increase the weight of one control mechanism over the other, by influencing their cognitive costs. According to one of the DMC postulates, limited reactive control capacities should be counterbalanced by greater proactive control to ensure control efficiency. Moreover, as the flexible weighting between reactive and proactive control is key for adaptive behaviors, we expected that maladaptive behaviors, such as risk-taking, would be characterized by an absence of such counterbalance. However, to our knowledge, no studies have yet investigated this postulate. In the current study, we analyzed the performances of 176 participants on two reaction time tasks (Simon and Stop Signal tasks) and a risk-taking assessment (Balloon Analog Risk Taking, BART). The post-error slowing in the Simon task was used to reflect the spontaneous individuals’ tendency to proactively adjust behaviors after an error. The Stop Signal Reaction Time was used to assess reactive inhibition capacities and the duration of the button press in the BART was used as an index of risk-taking propensity. Results showed that poorer reactive inhibition capacities predicted greater proactive adjustments after an error. Furthermore, the higher the risk-taking propensity, the less reactive inhibition capacities predicted proactive behavioral adjustments. The reported results suggest that higher risk-taking is associated with a smaller weighting of proactive control in response to limited reactive inhibition capacities. These findings highlight the importance of considering the imbalanced weighting of reactive and proactive control in the analysis of risk-taking, and in a broader sense, maladaptive behaviors.

## Introduction

In daily life, adapted motor behaviors are goal-directed. To achieve these goals, we must constantly process and integrate multiple competitive sources of information in order to readjust behaviors. Even for a casual daily activity, such as driving to the grocery store, the cognitive functions involved to achieve our goal are complex. They include keeping track of the grocery list and updating it in memory, retrieving and remembering the route to the destination, but above all, focusing on the road and monitoring drivers’ and pedestrians’ behaviors to adjust our own actions. The optimal coordination of all the involved cognitive processes is even more necessary in complex and unpredictable environments such as crowded streets. In such a situation, to ensure that our goal to stay safe until arriving to the grocery store is achieved, two control strategies can be adopted. The first is to be constantly alert of traffic information and to slow down when approaching the crowded area to more easily avoid potential accidents. The second, which is the opposite strategy, consists in not modifying our behavior when approaching the crowded area, but in relying on our ability to react when an accident is likely to occur (e.g., by braking if a pedestrian suddenly crosses the road). Although both strategies lead to the same goal, namely to avoid the accident, they differ in the way this goal is achieved. Investigating inter-individual differences in the implementation of these cognitive control strategies may be the key to gain a better understanding of adaptive behavior [1].

Cognitive control is a set of basic executive functions that guide and adjust goal-directed actions according to internal and external demands [2, 3]. According to the dual mechanisms of control framework [1], on the one hand, proactive control aims to anticipate future difficulties by facilitating the resolution of the competition between the multiple sources of information. To do so, it relies on the active maintenance of the goal in memory and the anticipatory selection of goal-relevant information to achieve it. Therefore, proactive control is costly as it implies working memory resources and sustained attention [1, 4]. A driver who turns down the radio to pay more attention to the road-relevant information engages proactive control resources. On the other hand, reactive control aims to resolve the competition at the time it occurs and consists in the retrieval of goal-relevant information when a difficulty is detected, leading to a novel decisional process and the late correction of erroneous behaviors [1, 4]. Reactive control is thus less costly than proactive control since it is recruited on an as-needed basis. The driver who brakes at the last minute if a pedestrian decides to cross illustrates reactive control. Therefore, proactive and reactive mechanisms rely on different temporal dynamics, but both use goal-relevant information in order to adjust goal-directed behaviors to external and internal demands.

Proactive and reactive control are two complementary but independent cognitive control mechanisms that co-exist in the cognitive control system [1]. They are not mutually exclusive as they are often simultaneously engaged during a cognitive control task to optimize behavioral outcomes [5, 6]. However, their recruitment to perform a task is weighted according to their respective cognitive costs, i.e., load of the cognitive resources needed for an optimal efficiency. Proactive control mechanisms rely on sustained attention to goal-relevant information and thus, highly consumes cognitive resources while enabling to resist attentional capture generated by distractors. In contrast, reactive control mechanisms rely on flexibility and inhibitory capacities and thus, enable fast adjustments to changes in the environment. Their drawbacks are that they are more sensitive to disturbances and thus, less robust [7]. Also, since they can take effect too late, i.e., only after the need for adjustment is detected, they are the last opportunity to successfully adjust actions. The weighted recruitment of the two control mechanisms depends, at least to a large extent, on these specific cognitive costs and benefits that are known to be modulated by both contextual and inter-individual characteristics [1, 4, 7]. It has been found that proactive control is favored by individuals with a high working memory capacity, as the cost induced by sustained attention is lighter for them compared to that experienced by those with a low working memory capacity [8, 9]. Older adults showing impairments in goal maintenance also favor the use of reactive control [10]. However, when these deficits have little or no impact on performance, for example in a low cognitive load condition, ageing was associated with a tendency toward proactive control [11, 12]. Indeed, the environmental context can also modulate the favored strategy by shifting it towards proactive or reactive control. High task-context load generally decreases the use of proactive control, as the goal-relevant information is too heavy to process continuously [11, 13]. On the contrary, a high motivational context favors the use of proactive control to ensure optimal efficiency of control [14, 15]. Therefore, the costs of the deployment of the control strategies vary as a function of contextual and inter-individual (e.g., personality, age, cognitive capacities) characteristics [e.g., 8, 9, 13, 16].

To our knowledge, no study has investigated whether potential limitations in reactive control capacities may be associated with a higher propensity to use proactive control. Indeed, if reactive control is insufficiently efficient, the successful online resolution of a competition between responses, i.e., before a response is executed, is uncertain. Therefore, to ensure the efficiency of the cognitive control system, proactive control should be strongly engaged to prevent the emergence of such competition. The weighted recruitment of proactive control mechanisms according to reactive control capacities would thus respect the principle of parsimony of cognitive systems, optimizing the efficiency of cognitive control while reducing its costs. Consistent with these statements, our general hypothesis was that individuals with effective reactive capacities would generally less rely on proactive control processes ensuring adapted behaviors without substantial cognitive cost. Conversely, individuals with low reactive capacities should be those who generally rely more on proactive control strategy ensuring adapted behaviors by maintaining the effectiveness of the cognitive control system despite the generated cognitive cost.

To test this hypothesis, two behavioral indices were chosen to reflect reactive and proactive control mechanisms. As reactive control rely on inhibitory capacities to correct actions in face of changes in the environment, we used the Stop Signal task to measure reactive control capacities through the Stop Signal Reaction Time (SSRT) [17, 18]. Indeed, the SSRT is a precise and reliable task evaluating the capacity to inhibit an ongoing but no longer appropriate response. Thanks to the adjustment of task difficulty to one’s performance, the SSRT is also more sensitive to inter-individual variability in a healthy sample than other simpler inhibition indices, such as the error rates in the Go/NoGo task [18]. Secondly, even if the literature does not suggest a large panel of behavioral measures of proactive control recruitment, the post-error slowing (PES) was considered in the current study as a satisfactory and reliable candidate to reflect the spontaneous use of proactive control [19]. Despite the ongoing debate on the involvement of control mechanisms in the PES [20, 21], the performance slowing after an error measured by the PES was showed to be associated with an increase in response caution [22]. More specifically, the slowing of RTs after an error might reflect the use of the error as a cue to proactively refocus attention on goal-relevant information. Thus, in the present study, SSRT and PES were used as indices for to reactive and proactive control mechanisms, respectively.

In the current study, we tested the general hypothesis postulating that individuals with low reactive capacities should rely more on proactive control strategy than individuals with effective reactive capacities (H1). Moreover, as adaptive behaviors rely on this flexible weighting of proactive and reactive control mechanisms [1], one could hypothesize that an inflexible weighting of the two control mechanisms would result in maladaptive behaviors (i.e., insufficiently controlled behaviors). Risk-taking is largely associated with many maladaptive behaviors such as substance abuse [e.g., 23, 24], eating disorders [e.g., 25–27], and aggression [28] and can be objectively measured with the Balloon Analog Risk Task [29]. Among the various questionnaires and experimental paradigms that can be used to assess risk-taking [30–32], the BART particularly benefits from a large literature on its relationship with real-life maladaptive behaviors such as drug use [33–35] and delinquency [36]. Our second hypothesis was that the effect of limited reactive capacities on the increased propensity to use proactive control would be less observed in individuals with high risk-taking propensity (H2). In other words, we hypothesized that higher risk-taking propensity would be associated with less proactive counterbalance as a function of reactive inhibition capacities.

## Method

### Participants

A total of 571 volunteers (300 males) with a normal or corrected-to-normal vision were recruited on several sites in France using ads on social networks. Ages ranged from 18 to 92 years (*M* = 36.79, *SD* = 16.91). Data were collected from May to July 2018, then from August 2018 to September 2019. Participants were evaluated on a battery of cognitive tests including the three tasks used in the present study, followed by an on-road driving test assessed by professional driving instructors. The results of the driving test were not used for the current analysis. Ethical approval for the current study was obtained from the ethics committee of the University of Lille (2017-9-S55). After application of exclusion criteria, which will be explained in more detail below, 176 participants were included for further analyses (98 men), the age ranged from 18 to 90 years (*M* = 33.68, *SD* = 15.77).

### Experimental tasks

#### Stop signal task

Participants were individually invited to perform a modified version of the classic Stop Signal task [17, 18] in which he/she was instructed to respond right or left, as fast and as accurate as possible, according to the direction of a white arrow which was presented at the center of a screen (Go trials). The response devices were composed of two buttons placed on vertical joysticks linked to a computer screen displaying the stimuli (60Hz refresh rate). He/she was also informed that in some trials, the arrow could turn red (Stop trials) and that this was the signal that they had to refrain from answering. Instructions were given orally and in writing on the computer screen to ensure proper understanding.

Each trial began with the presentation of a fixation cross for 300 ms (fixed foreperiod duration). Then, the left or right pointing arrow appeared at the center of the screen until the participant gave a response. In the absence of a response, the arrow disappeared after a delay of 1500 ms. Trials were separated by a blank screen lasting 500 ms. Therefore, the response-stimulus interval was 800 ms. In 25% of the trials (the Stop trials) and after a certain delay (the Stop Signal Delay, SSD), a Stop Signal appeared, which instructed the participant to withhold the response by inhibiting the engaged motor command. In our study, the arrow color-change to red illustrated this Stop Signal. The SSD, initially set to 200 ms, was adjusted trial-by-trial according to each participant’s performance. In a Stop trial, if the participant succeeded to inhibit in time his/her response, the SSD was increased by 50 ms. If the participant responded despite the presentation of the Stop Signal, the SSD was decreased by 50 ms. This tracking procedure was explained explicitly to the participants, as recommended by Verbruggen et al. [18], so that they would not be tempted to slow down their response times in order to beat the algorithm (i.e., they were warned that such errors were inevitable and that the difficulty of the task would adapt to their performance rate). A short training phase of 20 trials was implemented in order to familiarize participants with the task instructions and the response device. During training only, feedback was provided at the end of each trial for 500 ms (i.e., “Well done, correct response”—*“Bravo*, *bonne réponse”* for a correct response, “Incorrect response”—*“Réponse incorrecte”* for an error, “Try to be faster”—*“Essayez d’être plus rapide”* for an omission during a Go trial and “Try to stop”–“*Essayez de vous arrêter*” for a response despite the Stop Signal). The experimental phase of the Stop Signal task was composed of two blocks of 129 trials (258 trials) without any feedback and lasted approximatively six minutes.

The Stop Signal indicates that the current action is no longer adapted to task demands; thus, it triggers reactive control mechanisms to resolve the competition between the ongoing erroneous response and the correct response. Therefore, the Stop Signal reaction time (SSRT) was used as an index of the reactive inhibition capacity, which refers to the voluntary inhibition of an ongoing motor response [17, 37]. The SSRT was calculated following the integration method detailed by Verbruggen et al. [18], to take into account the variability of the RTs in the Go trials, Go omissions and the probability of successful Stop trials. The longer the SSRT, the more time is needed for the participant to stop the ongoing action and thus, the less efficient is the reactive inhibition.

#### Simon task

The participant was instructed to perform a modified version of the classic Simon task [38]. In this variant, he/she was required to respond as fast as possible to the shape of the stimuli (a white square or a white circle) that could appear on the right or left side of the fixation cross. The response devices were composed of two buttons placed on vertical joysticks linked to a computer screen displaying the stimuli (60Hz refresh rate). The participant was told that 5% of errors was accepted to ensure that he/she performed the task under time pressure according to the speed-accuracy trade-off postulate [39]. Instructions were given orally and in writing on the computer screen to ensure proper understanding.

Each trial began with a fixation cross, presented for 300 ms (fixed foreperiod duration). Then, the stimulus was displayed until a response was given. In the absence of a response, the stimulus disappeared after a delay of 1500 ms. Trials were separated by a blank screen lasting 500 ms. Therefore, the response-stimulus interval was 800 ms. The stimulus-response mapping created 50% of incongruent trials and 50% of congruent trials. A short training phase of 20 trials was implemented in order to familiarize participants with task instructions and response device. During training only, feedback was provided at the end of each trial for 500 ms (i.e., “Well done, correct response”—*“Bravo*, *bonne réponse”* for a correct response, “Incorrect response”—*“Réponse incorrecte”* for an error and “Try to be faster”—*“Essayez d’être plus rapide”* for an omission). The experimental phase of the Simon task was composed of two blocks of 129 trials each (258 trials), without any feedback, and lasted approximatively six minutes.

The Simon task was used to calculate the post-error slowing (PES), which refers to the lengthening of reaction times after the commission of an error compared to that observed after a correct response [40]. Following Pfister et al. [41] methodological guidelines to account for the pre-error speeding, the calculation of the PES followed the traditional method [40] (i.e., difference between the mean RT of correct trials directly following an error and the mean RT of correct trials directly following a correct response). On average, 218.44 (SD = 12.03) post-correct correct trials and 16.78 (*SD* = 5.05) post-error correct trials (range from 5 to 39 in the analyzed sample) were used in the calculation of the PES. The PES has been largely reported in studies using wide panel of paradigms and can be interpreted as a reallocation of executive attention towards goal-relevant information for the next trial to avoid persevering in error [20, 22]. Thus, in the current study, this measure was used as an index of the strength of proactive behavioral adjustment [19].

#### Balloon Analog Risk Taking task

In the Balloon Analog Risk Taking task [29], the participant was asked to virtually inflate 30 balloons by pressing a button to earn points. The response device was the same as for the other two tasks. However, the participant chose his/her dominant hand to press the button. Instructions were given orally and in writing on the computer screen to ensure proper understanding. The more inflated the balloon, the more a participant earned points. Each 500 ms of pumping gave one point. The participant was also informed that the balloons could explode at any time. Hence, the longer the participant pressed the button during a given trial, the greater risk the balloon had to explode. The predefined time before explosion ranged between 7 and 14 seconds (*M* = 10.0 s, *SD* = 1.8 s). If it exploded, the participant lost the cumulated points obtained in the current trial. Therefore, the participant had a choice: keep inflating the balloon to earn points but risking explosion, or stop inflating to earn less but keep the cumulated points. To challenge the participant and to promote risk-taking, the instructions set a goal score to exceed 400 points.

The mean duration time of the button-press for the unexploded balloons was used as an index of risk-taking propensity [Risk-Taking Index, RTI, 29]. A longer duration of the button press indicated a higher risk-taking propensity.

### Statistical analysis

The current study had two objectives. On the one hand, we aimed to explore the predictive value of reactive inhibition capacities (indexed by the Stop Signal Reaction Time–SSRT in ms) on the implementation of proactive behavioral adjustments (indexed by the post-error slowing–PES in ms). We expected that longer SSRT would predict greater PES (H1). On the other hand, we wanted to investigate potential modulations of the weighting of proactive control relative to reactive capacities by the risk-taking propensity (RTI, s). We expected that the higher RTI, the less SSRT would predict PES (H2). To do so, we used a mixed effect linear model (lme4 and lmertest packages on RStudio) [42, 43] to analyze the PES as a function of SSRT and RTI, both considered as fixed effects. To complete the model, we considered sex and age as random effects, since these variables are known to affect our variables of interest. Indeed, cognitive capacities, and in particular executive functions, are known to decrease with age. Moreover, men may take more risks than women [44–46]. Hence, the effects of sex and age on the three variables of interest were analyzed individually to orient the structure of random effects, using t-test and linear regression analyses, respectively.

Before calculating the indices of interest, reaction times inferior to 150 ms were removed. Then, the remaining RTs were filtered through a 2*SD interval calculated for each participant to remove individual performance outliers due to potential attentional lapses.

## Results

### Global analyses

The global analyses were performed on the entire sample (*N* = 571). In the Simon task, the mean correct reaction time was 511.55 ms (*SD* = 71.79) and the global error rate was 4.72% (*SD* = 2.62). Error rates for incongruent trials (*M* = 6.03%, *SD* = 3.75) were larger than for congruent trials (*M* = 3.41%, *SD* = 2.80), *t*(1055.2) = -13.35, *p* < .001, Cohen’s *d* = 0.79. There was also a significant effect of congruency on RTs (*M* = 27.30 ms, *SD* = 19.37), *t*(570) = 33.69, *p* < .001, Cohen’s *d* = 1.41. Reaction times in incongruent trials were longer (*M* = 525.50 ms, *SD* = 73.91) than in congruent trials (*M* = 498.20 ms, *SD* = 70.87), *t*(570) = 33.69, *p* < .001, Cohen’s *d* = 1.40. There was a significant slowing of RTs after errors (*M* = 57.57 ms, *SD* = 48.26), *t*(570) = 28.52, *p* < .001, Cohen’s *d* = 1.19. Reaction times in correct trials after an error (*M* = 566.43, *SD* = 98.08) were longer than reaction times in correct trials after a correct response (*M* = 508.85, *SD* = 71.23), *t*(570) = 28.52, *p* < .001, Cohen’s *d* = 1.19. In the Stop Signal task, the mean reaction time in correct Go trials was 578.96 ms (*SD* = 234.38) and the mean SSRT was 244.56 ms (*SD* = 91.37). Finally, the mean duration time of the button-press to pump balloons at the BART was 7.69 s (*SD* = 0.71). The mean of cumulated points was 356.47 (*SD* = 38.04).

### Application of inclusion criteria

Before testing the hypotheses, the inclusion criteria were applied to the overall sample to ensure that the participants included in further analyses had completed the tasks in accordance with the instructions.

We first applied the criterion of adequate performances in the Simon task. In this task, only participants with an error rate superior to 5% were selected to ensure performances under time pressure, mobilizing cognitive control resources, i.e., normal instructions for speed-accuracy trade-off [39]. This strategy was adopted to include participants that did complete the task as fast as they could, while complying with the given instructions, and making enough errors to calculate a reliable post-error slowing index. Among the global sample, the threshold of minimal 5% of errors included 193 participants. The highly-accurate participants (i.e., less than 5% of errors) were slower to perform the task (516,73 ms) than these less-accurate participants (501,31 ms), *t* = 2.36, *p* = .019, Cohen’s *d* = 0.22. Finally, 17 participants were removed as their Stop Signal performance did not meet the criterion of Verbruggen et al. [18], and had outliers SSRT.

### Analysis of the confounded variables

The effects of two potential confounded variables (i.e., age and sex) were tested on the included sample (*N* = 176). The effect of the discrete variable on the three variables of interest was analyzed using Student’s t-test (cf. Table 1). There were no significant effects of sex on PES, SSRT or on RTI, all *p*-values > .050.

**Table 1:** Means (M) and standard deviations (SD) of the post-error slowing (ms), the Stop Signal reaction time (ms) and the press duration (s) in the BART.

Variables of interest	All sample (*N* = 176)		Men (*N* = 98)		Women (*N* = 78)		*t*	*df*	*p*	Cohen’s *d*
	*M*	*SD*	*M*	*SD*	*M*	*SD*				
PES (ms)	54.12	41.17	57.31	40.4	50.23	42.04	1.13	162.21	.260	0.17
SSRT (ms)	271.93	53.5	269.18	48.72	275.38	59.1	0.75	148.36	.456	0.12
RTI (s)	7.78	0.71	7.71	0.67	7.88	0.74	1.56	157.38	.122	0.24

*Note*. PES = post-error slowing ; SSRT = Stop Signal reaction time ; RTI = Risk-Taking Index.

**Fig 1:**
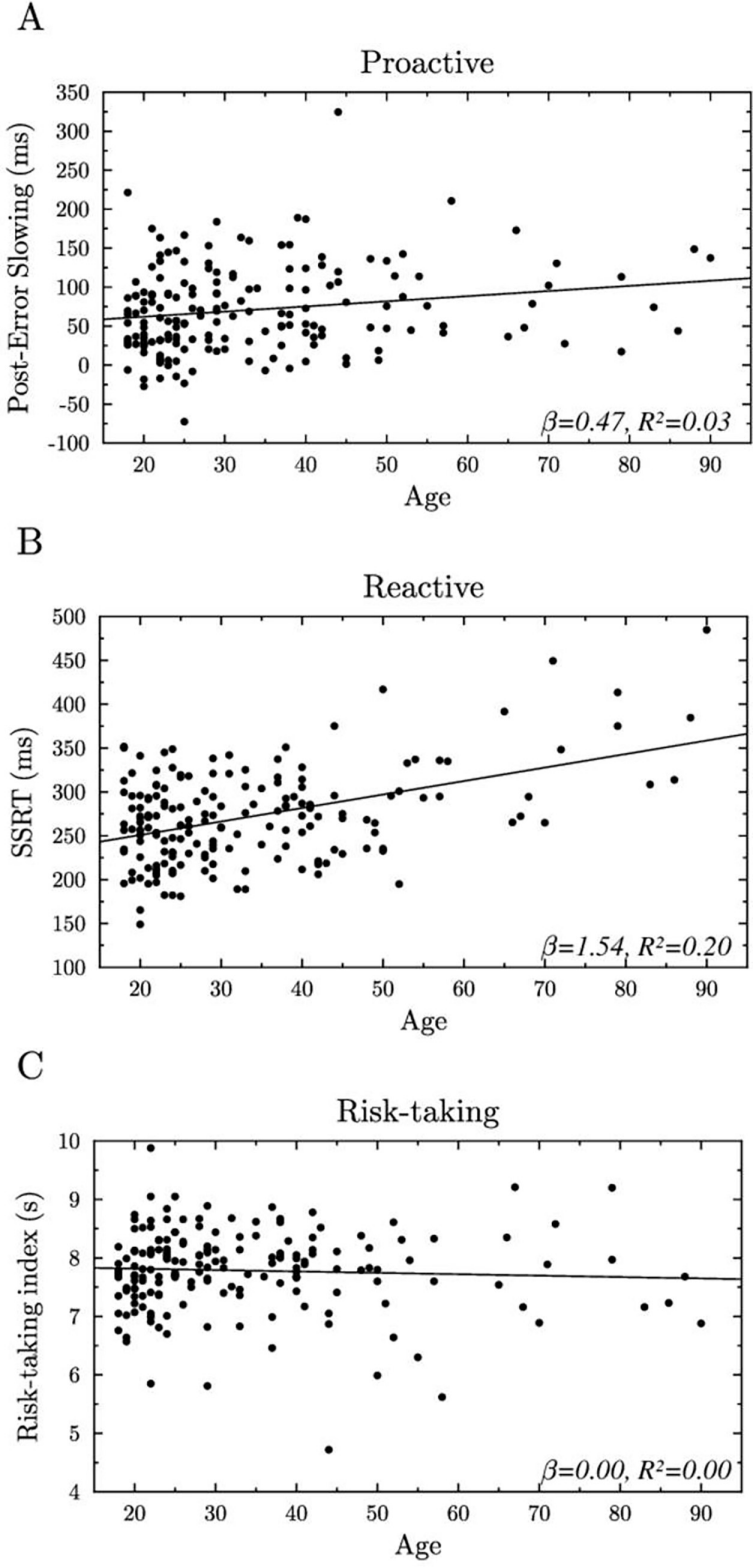
Effects of age on the three variables of interest. **(A)** the post-error slowing (ms), an index of proactive behavioral adjustments, **(B)** the Stop Signal reaction time (ms), an index of reactive inhibition capacity and on **(C)** the mean button press duration (s) the risk-taking index in the BART task.

The effect of the continuous variable on the three variables of interest was analyzed using simple linear regression. Results showed a significant effect of age both on the PES and on the SSRT, *β* = 0.47, *t*(174) = 2.43, *SE* = 0.19, *p* = .016, adjusted *R^2^* = 0.03 and *β* = 1.54, *t*(174) = 6.71, *SE* = 0.23, *p* < .001, adjusted *R^2^* = 0.20, respectively (see Fig 1A and 1B). However, there were no significant effects of age on the RTI, *β* = 0.00, *t*(174) = -0.70, *SE* < 0.01, *p* = .483, adjusted *R^2^* < 0.01 (see Fig 1C).

As age had an effect on both PES and SSRT (cf. Fig 1), age was set in the model as a random effect to control for its confounded influence. As sex had no effect on the variables of interest (cf. Table 1), sex was not added to the model.

### Modulation of proactive adjustments as a function of reactive inhibition and risk-taking propensity

**Fig 2:**
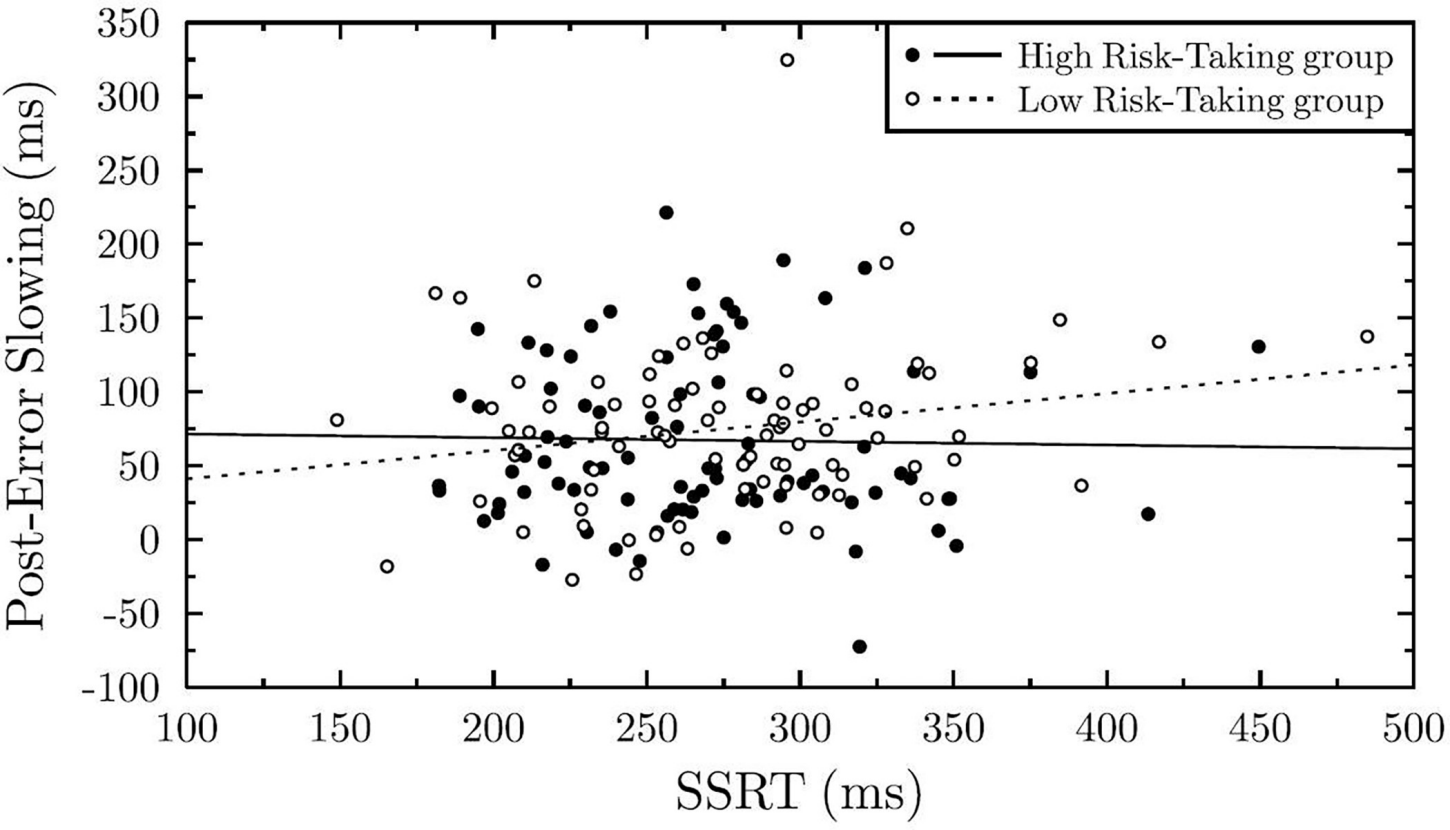
Proactive behavioral adjustments as a function of reactive inhibition capacity and risk-taking propensity. The SSRT (Stop Signal Reaction Time) was used as an index of reactive inhibition capacities. The PES (Post-Error Slowing) was used as an index of proactive behavioral adjustments. The risk-taking groups were created for visualization purpose only. We used the median of the RTI (Risk-Taking Index, the mean duration of the button-press at the BART task) distribution to categorize participants as high (*N* = 87) or low risk-takers (*N* = 86).

In the current study, we aimed at exploring the predictive value of the SSRT, and its interaction with risk-taking propensity (RTI) on the PES, while controlling for the effect of age. Longer SSRT significantly predicted greater PES, *β* = 1.36, *t*(77.94) = 2.16, *SE* = 0.63, *p* = .034. The RTI alone did not significantly predict the PES, *β* = 37.35, *t*(96.37) = 1.58, *SE* = 23.62, *p* = .117. However, results showed a significant interaction effect between SSRT and RTI on the PES, *β* = -0.17, *t*(82.17) = -2.08, *SE* = 0.08, *p* = .040. The greater the RTI, the less SSRT predicted PES (cf. Fig 2). This effect appeared to be robust. Indeed, when including participants whose performances on at least one of the two tasks did not meet the inclusion criteria (*N* = 571), we observed a similar significant interaction effect, β = -0.06, *t* = -1.98, *SE* = 0.03, *p* = .048.

## Discussion

The aim of our study was to determine whether reactive inhibition capacities could constrain a general tendency toward proactive control and whether the weakening of this effect would be associated with risk-taking propensity. We first hypothesized that an individual with inefficient reactive control capacities would have a higher tendency to use proactive mechanisms to guarantee adapted behaviors. Results showed that longer Stop Signal Reaction Time (SSRT) significantly predicted greater post-error slowing (PES), thus confirming H1. Secondly, we hypothesized that the propensity to take risks would alter the increased weighting of proactive mechanisms in response to limited reactive capacities. We observed that the higher the risk-taking index (RTI), the less the SSRT predicted PES, thus confirming H2. These findings suggest that higher risk-taking is associated with a smaller weighting of proactive control to counterbalance poorer reactive inhibition capacities. Imbalanced weighting between reactive and proactive control could be a fine-grained index for the detection of maladaptive behavior.

Cognitive control relies on processes that can be defined as part of either reactive or proactive mechanisms. These control mechanisms are independently engaged to handle conflicting situations [1] and are associated with different cognitive costs and benefits [7]. The balanced weighting of proactive and reactive control is a function of inter-individual and contextual characteristics and favors one control mechanism over the other to limit cognitive costs and optimize behavioral outcomes. Interestingly, some studies have reported the effects of inter-individual factors (e.g., cognitive capacities, age, personality traits) on the preferential use of one mechanism over the other [e.g., 8–10, 16]. Two previous studies have demonstrated a tendency towards greater use of reactive control when proactive control was limited by poor working memory capacities, on which active maintenance of goal-relevant information is based [8, 9]. To the best of our knowledge, the literature had not yet addressed the opposite effect, i.e., the effect of limited reactive capacities on the tendency to use proactive control. In the current study, we thus hypothesized that the involvement of proactive behavioral adjustments would be increased in individuals with inefficient reactive inhibition capacities. Our results were in favor of this hypothesis. Indeed, the SSRT predicted the PES: larger SSRT (i.e., poorer reactive inhibition capacities) were associated with larger PES (i.e., stronger proactive behavioral adjustments after an error). Our current findings were in line with previous research that reported larger PES in individuals with lower cognitive resources, suggesting proactive control as a compensatory mechanism [47, 48]. Nonetheless, other studies have reported opposite results: impaired older adults showed less reliance on proactive control [10], which could seem inconsistent with our present findings reporting an increase in the PES with age and replicating the literature [47]. However, it is important to note that the PES reflect one proactive process of a more broad cognitive mechanism. Other proactive-related cognitive capacities, such as proactive inhibition, working memory or sustained attention, might be impaired in older adults, leading to the global less reliance on proactive mechanism reported in Paxton et al. [10]. Moreover, in our study, the task was performed in a low cognitive load context, that might have been not sufficient to reveal age-related cognitive deficits, explaining why elderly individuals could still rely on proactive control processes for this particular task [11, 12]. To complement these findings, future researches should investigate the balance between reactive capacities and the recruitment of proactive processes through age in a more cognitive challenging context.

The present study also aimed at investigating whether maladaptive behaviors could be associated with a maladapted weighting in the recruitment of control mechanisms according to the environmental context, and in particular in the current study, to one’s own capacities. The second aim was thus to investigate the influence of risk-taking propensity on the observed weighted recruitment of proactive and reactive mechanisms. Our results showed that higher risk-taking propensity was associated with a smaller increase in post-error slowing in response to limited reactive inhibition capacities. In other words, larger SSRT predicted to a lesser degree the PES with an increase in risk-taking propensity. The imbalanced weighting between the two control mechanisms with high risk-taking propensity reduced the global cost of control, since less proactive and costly behavioral adjustments were engaged. However, this strategy may also reduce control efficiency. Therefore, these findings have direct applicability in the general population, for which the understanding of the emergence of maladaptive behaviors is crucial. Currently, maladaptive behaviors are mostly viewed as resulting from the lack of inhibition, but numerous studies are more recently showing an absence of association between disinhibition and various maladaptive behaviors [49–52]. The current findings suggest that the assessment of the adapted weighting of reactive and proactive control mechanisms could be useful to create a finer-grained index of an individual’s tendency to adopt maladaptive behaviors. At the behavioral level, the efficient recruitment of proactive control mechanisms could hide and compensate for limited reactive inhibition capacities. An individual knowing that he/she is unable to stop the car in time to avoid an accident should slow down to efficiently drive. If such strategy is adopted then, this individual should not be considered as an at-risk individual. However, the same individual would be at-risk in another context requiring the use of reactive control. On the other hand, an excessive recruitment of proactive control mechanisms in individuals whose reactive capacities are efficient would generate a cognitive overload for the control system. Such individuals would be characterized by high cognitive fatigue, resulting in decreased performance [53].

In the current study, three specific behavioral measures were chosen to assess inter-individual variability in the spontaneous tendency to use proactive control, reactive control capacities and risk-taking propensity. However, as already mentioned, the literature offers other indices that could have also been used to differently evaluate reactive capacities and the general tendency to use proactive control, as other ways to assess risk-taking. Therefore, the performed analysis of their relationships reported in the current paper is one way to test our hypotheses. In future studies, alternative indices could be used to investigate different facets of reactive and proactive control and hence, consolidate the reported findings. Firstly, it would be interesting to use an index of reactive inhibition that is internally generated, such as reflected in the partial-errors and the correction ratio [54, 55]. Indeed, the SSRT reflects an externally triggered reactive inhibition, whereas the correction of partial-errors operates without an external cue. Secondly, the PES is as a short-term proactive adjustment as it decays with inter-trial interval [56]. Hence, the PES is not an index of long-term strategic adjustments that are reflected in the global slowing of reaction times as a function of the Stop likelihood in the Stop Signal task [57]. Also, the proactive behavioral index calculated on the performances at the AX-CPT task would also be an interesting way to measure the reliance on proactive control strategy as a function of reactive capacities. The analysis of the relationship between partial-error correction ratio and the global slowing of reaction times or the proactive behavioral index could be a further step in the understanding of the weighted recruitment of reactive and proactive control mechanisms in healthy individuals. Finally, other maladaptive behaviors than risk-taking could be considered. The relationship between the imbalanced weighting of control mechanisms and disadvantageous decision-making, evaluated in the Delay Discounting task [58], could support our findings.

## Conclusions

The present study demonstrated that reactive inhibition capacities predict the general tendency towards proactive control, but this effect is reduced with higher risk-taking propensity. To our knowledge, these are the first empirical arguments that confirm a stronger reliance on proactive control in individuals with low reactive control capacities. Also, poor inhibition capacities may not be sufficient enough to index the tendency to adopt maladaptive behaviors, but the absence of a proactive counterbalancing could be a finer-grained index to identify at-risk individuals.
